# Comment on: “Interpretable machine learning model for predicting early recurrence of pancreatic cancer: integrating intratumoral and peritumoral radiomics with body composition”

**DOI:** 10.1097/JS9.0000000000003323

**Published:** 2025-08-22

**Authors:** Yuqin Zhang, Wei Tang, Yue Deng

**Affiliations:** Department of Radiology, Suining Central Hospital, Suining, Sichuan Province, China

*Dear Editor,*

We have read with great interest the article by Wu *et al*^[1]^ titled “Interpretable Machine Learning Model for Predicting Early Recurrence of Pancreatic Cancer: Integrating Intratumoral and Peritumoral Radiomics with Body Composition.” Through innovative multimodal data integration and explainable artificial intelligence technology, this study has made significant progress in the field of prognostic prediction of pancreatic ductal adenocarcinoma (PDAC), providing new insights into solving the critical challenge of early recurrence prediction.

The main strengths of this study are as follows. First, the authors adopted a multimodal approach, integrating intratumoral and peritumoral radiomic features, CT-quantified body composition parameters, and clinicopathological factors, which is notably innovative. By comparing six machine learning algorithms, the random forest-based intra-peri-radiomics model showed robust performance, with Area Under the Curve (AUC) values of 0.865, 0.849, and 0.839 in the training, internal validation, and external validation cohorts, respectively. After further incorporation of clinicopathological factors, the combined model performed better, with AUCs of 0.936, 0.899, and 0.884, respectively. This demonstrates the synergistic value of combining imaging features and clinical data to capture the complex biological mechanisms of PDAC recurrence. Second, the application of the SHapley Additive exPlanations (SHAP) analysis enhanced the interpretability of the model. By quantifying the contribution of each feature, key predictive factors were identified, including radiomic features, adjuvant therapy, CA199, lymphovascular invasion, and visceral fat index (VFI), effectively addressing the “black box” problem of machine learning. This transparency not only enhances clinicians’ trust, but also provides a basis for risk stratification and individualized treatment decisions. The decision curve analysis further verified its clinical applicability, indicating that the model can yield net benefits across a wide range of risk thresholds.

Notably, this study addressed the limitations of previous research, which either relied solely on intratumoral features or lacked external validation, through measures such as a multicenter design, rigorous validation procedures, inclusion of peritumoral radiomic features, and incorporation of body composition parameters—an often-neglected but biologically significant factor.

Despite these strengths, several aspects require further exploration and improvement. First, as noted by the authors, the retrospective study design and static assessment of radiomic features have limitations. Future multicenter prospective studies could validate the model’s real-world effectiveness, while incorporating dynamic changes in radiomic features and body composition during treatment (e.g., after chemotherapy), which may further optimize the predictive performance.

Second, the current radiomic features are derived only from the portal venous phase of CT, without covering other phases or imaging modalities (e.g., MRI and PET-CT), which may miss information related to tumor metabolism and hemodynamics. Subsequent studies could supplement features from MRI and PET-CT metabolic parameters, and combine them with CT radiomics to enhance the ability to capture tumor heterogeneity.

Third, although the study clarified the predictive contribution of each factor through SHAP analysis, it failed to establish links with the tumor microenvironment and molecular mechanisms, which to some extent limits its clinical translation value. Future research could incorporate genomic (e.g., KRAS mutation) and proteomic (e.g., circulating tumor DNA) data to improve the model, and explore the correlations between radiomic features (e.g., texture features, volume features) and KRAS mutations, ctDNA levels, as well as the associations between body composition parameters such as VFI and the expression of cytokines such as IL-6, thereby revealing potential imaging-biomarker relationships.

In conclusion, the study by Wu et al. made valuable contributions to PDAC prognostic assessment. This work not only advances the development of predictive models but also emphasizes the importance of model interpretability in translating machine learning into clinical practice. We highly appreciate the authors’ rigorous methodology and clinically meaningful research findings, and this model will provide important support for formulating postoperative management strategies and improving prognosis in high-risk patients.

## Data Availability

All data analyzed or generated during this study are included in this article.

## References

[1] Wu L Cen C Ouyang D. Interpretable machine learning model for predicting early recurrence of pancreatic cancer: integrating intratumoral and peritumoral radiomics with body composition. Int J Surg 2025:10–97. 10.1097/JS9.0000000000003078 PMC12626599 40717595

[2] Agha RA Mathew G Rashid R. Transparency in the reporting of Artificial INtelligence: the TITAN guideline. Prem J Sci 2025;10:100082.

